# Trauma transfers to a rural level 1 center: a retrospective cohort study

**DOI:** 10.1186/s13032-016-0031-z

**Published:** 2016-01-19

**Authors:** Sumeet V Jain, Castigliano M Bhamidipati, Robert N Cooney

**Affiliations:** Department of Surgery, SUNY Upstate University Hospital, 750 East Adams St, Syracuse, NY 13210 USA

**Keywords:** Trauma transfers, Epidemiology, Cost, Healthcare access

## Abstract

**Background:**

The regionalization of trauma care, the Emergency Medical Treatment and Active Labor Act of 1986, the advent of Accountable Care Organizations and bundled payments have brought Level 1 trauma centers (TC) to a new crossroads. By protocol, injured patients are preferentially transferred to designated TCs when a higher level of care is indicated. Trauma transfers frequently come during off hours and may not always appear to be related to injury severity. Based on this observation, we hypothesized patients transferred from regional hospitals to Level 1 TCs would have lower injury severity scores (ISS) and unfavorable payor status.

**Methods:**

We queried our TC registry to identify trauma transfers (TTP) and primary trauma patients (PTP) treated at our level 1 TC between 2004 and 2012. Demographics, payor status, length of stay (LOS), injury severity score (ISS), and discharging service were compared.

**Results:**

5699 TTP and 11147 PTP were identified. Uninsured patients comprised 11 % (*n* = 602) of TTP compared with 15 % (*n* = 1,721) of PTP (*P* < 0.0001). Surprisingly 52 % of TTP were Medicare or HMO (*n* = 3008) beneficiaries, versus 42 % of PTP being Medicare or HMO (*n* = 4614) recipients (*P* < 0.0001). Patients were discharged predominantly by neurosurgery and orthopedic surgery (i.e.: General Adult and General Pediatric comprised <50 % of discharges) for all trauma admissions. Adult and Pediatric Trauma services accounted for 29 % (*n* = 1674) of TTP versus 45 % of PTP (*n* = 5045) discharges (*P* < 0.0001). Mean Injury Severity Score of TTP was found to be 11.5 ± 0.11, in comparison to 11.6 ± 0.11 in PTP (*P* = 0.42), while mean LOS was 5.6 ± 0.1 days for TTP and 5.9 ± 0.1 days for PTP (*P* = 0.06).

**Conclusions:**

These data suggest designated trauma centers should continue to encourage and accept appropriate transfer of trauma patients for surgical subspecialty care. The perception trauma transfers increase institutional fiscal burden is unsubstantiated.

## Background

Organized state and regional trauma systems have changed the delivery of care for injured patients [1, 2]. Severely injured and “high-risk” patient populations are commonly transferred from smaller hospitals with fewer resources to regional trauma centers for optimal care. Regional trauma systems are based on military models to provide timely and appropriate treatment of trauma patients [3]. Numerous studies have demonstrated implementing an organized system of trauma care can significantly reduce mortality of injured patients [3–8]. Consequently, there are organized efforts to develop robust, collaborative trauma networks nationwide. Ideally, hospitals participating in national and state trauma systems would transfer patients to regional trauma centers based on need and not financial status. To help prevent this Congress passed the EMTALA in 1986 based on reports of “patient dumping”, or the practice of transferring unstable patients due to financial undesirability [9–11]. The object of EMTALA was to ensure universal access to emergency services by outlining specific obligations for all hospitals, to standardize patient care irrespective of patient factors. EMTALA specifically states a transferring hospital is responsible for minimizing a patient’s risk as much as possible and that the receiving hospital must accept transfers if there are qualified personnel and space available. By extension, regional Level 1 trauma centers were mandated to accept trauma transfer patients for definitive therapy in a regionalized system.

Several studies have postulated the implementation of EMTALA in conjunction with regionalized trauma systems have led to increased numbers of unnecessary patient transfers to tertiary care facilities based on undesirable insurance status [12–16]. The results have been mixed with some showing a discrepancy in the payor status of transfers and others showing an identical mix [12–16]. Being uninsured has been linked to decreased access to post-trauma care, as well as increased risk of penetrating trauma injury [17, 18]. Lack of insurance is also associated with increased morbidity and greater cost of care when compared with insured patients with similar injury mechanism [19–22]. As federal and state support of safety net hospitals and patients has decreased, the financial burden of poorly insured patients has received more scrutiny. A specific concern of tertiary centers is that they do not receive reimbursement for uninsured patients and therefore incur the extra costs associated with caring for this cohort.

Most of the studies looking at trauma transfers have either focused on subspecialty populations, have compared the transferred trauma population to admissions at transferring institutions or had relatively small patient numbers [12–16]. Our study is the first to examine all comers in a rural regionalized trauma center on a large scale. Previous studies looking at payor status within the subspecialist population suggest payor status is worse in the transferred group. Based on these results and our subjective experience, we hypothesized patients transferred from outside institutions would have an unfavorable payor status and lower injury severity compared to primary trauma patients. However, our results indicate that payor status and injury severity are similar in the transferred patient population, and that the primary reasons for transfer are increased access to subspecialist care and high-risk patient populations, especially at the extremes of age.

## Methods

### Study design and population

**Fig. 1:**
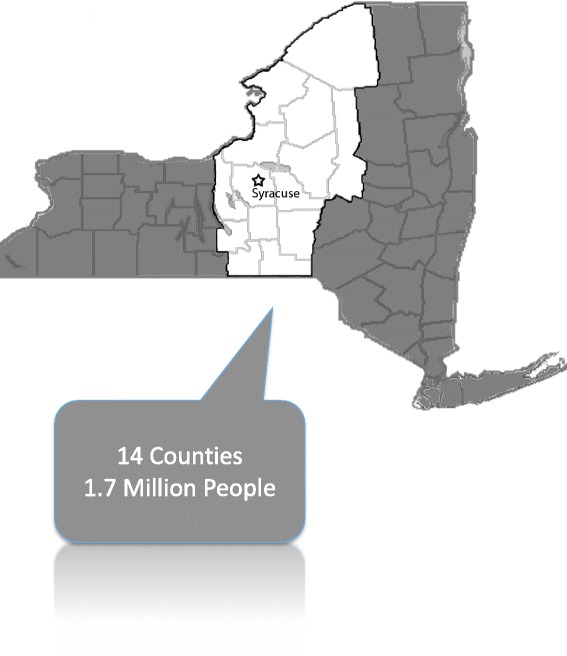
Catchment Area of SUNY Upstate Medical University. A map demonstrating the counties and population responsible for transfers to Upstate Medical University in our regionalized trauma system

Our hospital is an American College of Surgeons Verified Level 1 Trauma Center for adult and pediatric trauma. It is the primary trauma center for Syracuse, NY and Onondaga County, caring for more than 1200 trauma cases per year. In addition, our trauma center serves as a referral resource for injured patients cared for at lower level trauma centers in a 14 county region supporting a population of 1.7 million (Fig. 1). For over a decade our hospital has maintained a database of all trauma patients treated at our institution. This database is populated retrospectively and concurrently from the electronic medical record and repopulated with missing data. Data inclusion criteria are based on the National Trauma Data Bank and the NY State data dictionary regarding trauma registries. Data is validated based on American College of Surgeon’s requirements using random and focused samples as well as re-abstraction of charts. We completed a retrospective analysis from this database. The data was divided into transfer trauma patients (TTP), e.g. patients who were transferred from referring hospital for trauma care, and primary trauma patients (PTP), or those who arrived to our hospital first from the site of injury. All patients were included for analysis. Incomplete data was marked “unknown” where appropriate. This study was approved by the Institutional Review Board at SUNY Upstate Medical University, Syracuse, NY.

### Outcome measures

Patient factors including: age, gender, mechanism, injury severity score (ISS), and payor status were examined [23]. Institutional factors including: length of stay (LOS), discharging service, and disposition from ED were evaluated. Because of our interest in financial status and injury severity, our primary outcomes were payor status, ISS, and LOS. Secondary features of interest included the service that discharged the patients and age distribution. Payor status categories were streamlined by combining “self-pay” and “uninsured” categories, and combining multiple healthcare management organizations and Blue Cross Blue Shield into a “managed care” category. In analyzing age distribution, age categories were defined as follows: infants (0–3), young children [4–9], pre-teens [10–13], teenagers [14–17], young adults (18–49), middle-aged adults (50–64), older adults (65–79), and elderly adults (80+).

### Statistical analysis

Continuous variables were compared using an unpaired student’s *t*-test, and categorical variables were compared using chi-squared (*χ*^2^) test. Statistical analysis was performed using JMP^®^ 10 (Cary, NC) and GraphPad QuickCalcs™ (La Jolla, CA).

## Results

### Demographics

During the nine-year time period from 2004 to 2012, we treated 5699 TTP and 11147 PTP patients. Demographics of the study populations were similar. The average age (in years) of the TTP population was 42 ± 0.4 years compared with 39 ± 0.2 years in the PTP population (Table 1, *p* < 0.0001). Male patients made up 64 % of TTP vs. 68 % of PTP (*p* < 0.0001). Additionally, TTP had a much higher proportion of blunt injury compared with PTP (96 % vs. 85 %, *p* < 0.0001).

**Table 1:** Comparison of Trauma Primary vs. Transfer Patients

	Primary (*n* = 11147)	Transfer (*n* = 5699)	*p*-value
Age	39.0 ± 0.22	42.0 ± 0.36	<0.0001
Male	68 %	64 %	<0.0001
Blunt Injury	85 %	96 %	<0.0001
Injury Severity Score	11.6 ± 0.11	11.5 ± 0.11	0.4234
Length of Stay	5.6 ± 0.09	5.9 ± 0.11	0.06
*Student’s *t*-test			

### Payor status

**Fig. 2:**
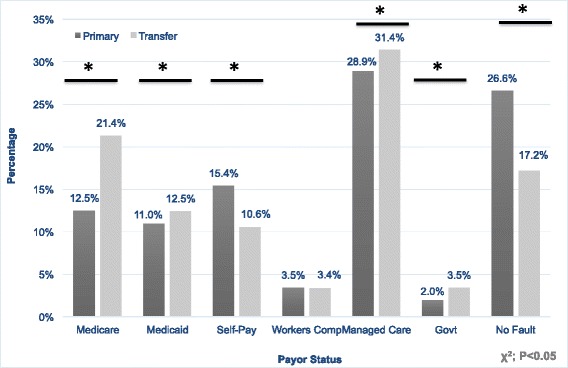
Payor Status of Primary Trauma Patients versus Trauma Transfer Patients. A breakdown of the proportion of primary trauma patients versus trauma transfer patients with specific payor categories. * = *p* < 0.05

When analyzing payor status, we noted a higher proportion of TTP with Medicare (21 % vs. 13 %, *p* < 0.0001) and managed care (31 % vs. 29 %, *p* = 0.0006) payors compared with PTP. TTP also had less self-pay (11 % vs. 15 %, *p* < 0.0001) and no fault coverage (17 % vs. 27 %, *p* < 0.0001). Medicaid coverage was slightly higher in TTP (12 % vs. 11 %, *p* = 0.0048; Fig. 2).

### Injury severity and length of stay

**Fig. 3:**
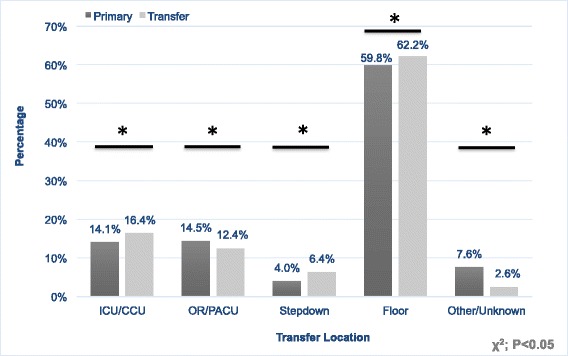
ED Disposition of Trauma Primary versus Transfer Patients. A comparison of the transfer location from the emergency department of trauma primary and trauma transfer patients. * = *p* < 0.05

TTP patients required more intensive care unit (ICU; 16 vs. 14 %, *p* < 0.0001), stepdown (6 % vs. 4 %, *p* < 0.0001), and floor admissions (62 % vs. 60 %, *p* = 0.0029) than PTP. However, TTP also required operations less frequently (12 % vs. 14 %, *p* = 0.0003; Fig. 3). Despite the increased rate of ICU admissions in trauma transfers, ISS was not significantly different between TTP (11.5 ± 0.11) and PTP (11.6 ± 0.10; *p* = 0.13; Table 1), nor was mean LOS significantly different (5.9 ± 0.11 vs. 5.6 ± 0.09; *p* = 0.0551).

### Age distribution

**Fig. 4:**
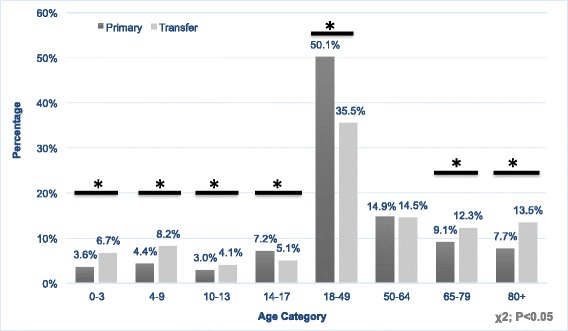
Age Distribution of Trauma Primary versus Transfer Patients. A comparison of the proportion of patients of specific age groups between trauma transfer patients and primary trauma patients. The comparison includes four groups within ages 0–17, representing the pediatric population and four groups above age 17 representing the adult population. * = *p* < 0.0001

TTP had a greater proportion of patients in the extremes of age (Fig. 4). The proportion of TTP in the infant (0–3) age range was 7 % vs. 4 % in PTP group, and in the young child [4–9] age range TTP was 8 % vs. 4 % in the PTP group. In addition, TTP had 26 % in the older adult category (>65) versus 17 % in the PTP group (*p* < 0.0001). Overall, 40 % of the TTP population was above the age of fifty and 25 % less than eighteen, compared with 32 and 18 % respectively in the PTP population.

### Discharging service

General Adult Trauma comprised less than 50 % of discharges in both TTP and PTP groups. However, discharges by General Adult Trauma were significantly lower in TTP than in PTP (27.0 % vs. 43.5 %; *p* < 0.0001). In fact, General Adult Trauma comprised the largest proportion of PTP discharges. In contrast, the majority (55.1 %) of the TTP patients were discharged by a subspecialty surgery service – namely orthopedics, neurosurgery, and otolaryngology – compared with of PTP (37.2 %; *p* < 0.0001; Table 2).

**Table 2:** Discharging Service: Trauma Primary vs. Transfer

	Primary	Transfer	*p*-value
Trauma	43.5 %	27.0 %	<0.0001
Pediatric Trauma	1.8 %	2.4 %	0.0120
Orthopedic Surgery	27.3 %	33.5 %	<0.0001
Neurosurgery	6.6 %	15.9 %	<0.0001
Otolaryngology	2.6 %	4.8 %	<0.0001
Other Surgery	0.7 %	0.9 %	0.3110
Nonsurgical	16.5 %	14.9 %	0.0054
Unknown	0.9 %	0.6 %	0.0248
*χ* ^2^ Test			

## Discussion

The goal of this study was to determine whether injured patients are being transferred from referring facilities inappropriately based primarily on financial status. If this were true, it would create an unnecessary financial burden on regional trauma centers. Therefore, changes in our trauma transfer protocols might be necessary to reduce this burden and provide better care. However, contrary to our hypothesis, TTP had a higher proportion in more favorable insurance categories, namely Medicare and Managed Care, and decreased proportion in the uninsured or self-pay population. Collectively these data suggest payor status is not a major determinant in the decision to transfer injured patients in upstate New York. This begs the following questions: “why does payor status show these discrepancies between these patient populations and what are the determinants for transfer?” There are several key factors that could address this discrepancy. First of all, the age distribution of TTP gravitates more towards the extremes of age than the PTP group, including a larger elder adult population that are covered by Medicare. Second, TTP has a decreased proportion of penetrating trauma, a population generally accepted to be underinsured [17]. Finally, more TTP were discharged by subspecialty services compared with PTP.

Analysis of our data suggests age is an important factor affecting transfer. It is well known that populations at extremes of age are much more likely to suffer morbidity from traumatic injuries and benefit from early transfer to designated trauma centers [24–29]. Pediatric trauma patients have been shown to have higher in-hospital mortality, length of stay, and cost of care in adult hospitals than pediatric-centered hospitals [25]. Additionally, increased age (>65 years old) is also a risk factor for the development of multiple organ failure morbidity from traumatic brain injury and overall morbidity and mortality [29–31]. The increased proportion of elder individuals in the TTP population also helps explain the larger number of Medicare beneficiaries in that population.

Surgery subspecialist availability can be problematic even at level 1 and 2 centers, let alone more regional referring institutions. For example, hand and microvascular call is inconsistent at level 1 and 2 trauma centers and one study in Cook County found that neurosurgical services had decreased across the board except at academic medical centers, as of 2008 [32, 33]. Given the significantly increased proportion of subspecialty surgical discharges, it appears at least one impetus for transfer was decreased subspecialty availability at referring hospitals and subsequent need for transfer for injuries requiring subspecialist surgical care.

There are several limitations to this study, as well as directions that can be further explored within our database. For one, most of the papers that cited a difference in payor status examined surgery subspecialties, especially neurosurgery and orthopedics [12–14, 34]. Consequently, subgroup analysis can be performed within these populations to see if there are different trends within the subspecialist service transfers compared with all transfers as a whole, and the primary trauma population. This analysis would be challenging because even if ISS or payor status were lower, it would be difficult to determine whether a transfer is based on subspecialist availability. There are other factors which could be analyzed such as ICU length of stay, mortality, ethnicity, and disposition from the hospital to name a few. Also, our data could be compared with the statewide database to compare the transferred patients with trauma patients who were not transferred. Finally, we are a level 1 trauma center in the middle of a primarily rural environment, and so our findings may not generalize well to a more urban population.

Our data suggest the general population of trauma transfer patients is insured, has similar injury acuity to our primary trauma patient population, and consists of extremes of age. Based on this observation, it seems likely the primary motivation for transfer is the need for subspecialty surgical care and not unfavorable insurance status. In the context of the Affordable Care Act, we are likely to see several changes in the landscape of medicine. First of all, the uninsured population will decrease, as more people are able to get access to insurance, primarily from increased Medicaid beneficiaries and decreased self-pay individuals because nonelderly Medicaid enrollment is estimated to increase by one-third [35] . This has already been described in New York State, with the expansion of Medicaid specifically, within the past decade before the Affordable Care Act (ACA) and resultant increase of Medicaid patients within subspecialty clinics after Medicaid expansion [36]. In addition, the implementation of mandatory health insurance has been tried in other states. Universal health insurance is associated with a global decrease in hospital LOS an associated increase in home health services and no change in mortality [37]. Therefore, we might expect the TTP and PTP populations to both experience an increase in reimbursement and decreased associated cost. This will not fully offset the losses incurred by tertiary care facilities. A large proportion of the cost of trauma care is due to the high standby costs associated with continuous coverage at Level 1 trauma centers and the significant costs of trauma program administration and performance improvement activities. These costs are not reimbursed by third party payors regardless of how much trauma is received. The ACA should, however, help mitigate the financial burden that tertiary trauma centers incur by allowing some reimbursement of patients where there was none [38]. Nevertheless, our results indicate that the perception that trauma transfers increase fiscal burden is unsubstantiated.

## Conclusions

Injured patients are transferred due to subspecialist availability and extremes of age, and not for financial reasons. In the context of known benefits to morbidity and mortality from transfer to a level I or II trauma center and further fiscal improvement with the ACA, there is a need for further development of a well-organized trauma network with directed and appropriate transfer to adequately care for the nation’s trauma population.
